# Bladder Cancer at the time of COVID-19 Outbreak

**DOI:** 10.1590/S1677-5538.IBJU.2020.S107

**Published:** 2020-07-27

**Authors:** Francesco Esperto, Karl H. Pang, Simone Albisinni, Rocco Papalia, Roberto M. Scarpa

**Affiliations:** 1 Campus Bio-Medico University of Rome Department of Urology Rome Italy Department of Urology, Campus Bio-Medico University of Rome, Rome, Italy; 2 University of Sheffield Academic Urology Unit Sheffield United Kingdom, UK Academic Urology Unit, University of Sheffield, Sheffield, United Kingdom, UK; 3 Université Libre de Bruxelles University Clinics of Brussels Department of Urology Brussels Belgium Department of Urology, University Clinics of Brussels, Hôpital Erasme, Université Libre de Bruxelles, Brussels, Belgium

**Keywords:** Urinary Bladder Neoplasms, BCG Vaccine, COVID-19 [Supplementary Concept]

## Abstract

The COVID-19 outbreak has led to the deferral of a great number of surgeries in an attempt to reduce transmission of infection, free up hospital beds, intensive care and anaesthetists, and limit aerosol-generating procedures. Guidelines and suggestions have been provided to categorize Urological diseases into risk groups and recommendations are available on procedures that can be or cannot be deferred. We aim to summarise updates on diagnosis, treatment and follow up of bladder cancer during the COVID-19 outbreaks.

## INTRODUCTION

The coronavirus disease 2019 (Covid-19) pandemic caused by the severe acute respiratory syndrome coronavirus-2 (SARS-CoV-2) has had major effects on individuals and healthcare systems ( 1 ). The virus was detected in Wuhan, China in December 2019 and as of May 10 2020, there are over 4.1 million cases and over 280,000 deaths worldwide ( 2 ). Protocols have been derived to limit hospital access and reduce services in an attempt to reduce transmission of infection, free up hospital beds, intensive care, anaesthetists and limit aerosol-generating procedures.

Urological diseases have been categorised into risk groups and recommendations are available on procedures that can be or cannot be deferred ( 3 , 4 ). Out-patient consultations are preferred to be performed through telemedicine. A recent study of 399 urology patients showed that 63.2% were eligible for telemedicine and 84.7% preferred a telemedical consultation during the COVID-19 period ( 5 ).

There are certain factors that affect the choice of different urological procedures such as the need for post-operative intensive care, the need for blood products and cardiovascular or respiratory co-morbidities. Patients with COVID-19 and multiple co-morbidities tend to have poorer outcomes ( 6 , 7 ).

Here, we discuss the impact and changes imposed on bladder cancer (BC) management.

### Bladder cancer epidemiology and classification

Bladder cancer is the 10th most common cancer worldwide, with an estimated 549,000 new cases and 200,000 deaths. Both the incidence and mortality is higher in men ( 8 ). Interestingly, men are more affected with COVID-19 and are more likely to get more severe disease ( 9 ). Tobacco smoking and occupational exposure to carcinogens are the factors with the highest attributable risk ( 10 ). However, there is increasing evidence to suggest the role of genetic polymorphism. The Cancer Genome Atlas (TCGA) provided molecular characterisation of BC based on somatic changes, with FGFR3 and KRAS implicated ( 11 ).

Approximately 75% of BC is non-muscle invasive (NMIBC) and include disease confined to the mucosa, pTa, carcinoma in situ (Cis) or to the submucosa, pT1. Muscle invasive BC (MIBC) accounts for 25% of BC diagnosed. The WHO grading system categorise BC into papillary urothelial neoplasm of low malignant potential (PUNLMP), low-grade (LG) and high-grade (HG) papillary urothelial carcinoma. Urothelial cell carcinoma (UCC) is the most common histological type. A subgroup of variants with worse prognosis has been described, which include micropapillary UCC, nested variant and microcystic UCC, plasmacytoid, small-cell carcinoma, sarcomatoid and the presence of lymphovascular invasion (LVI) ( 12 ). Stratification of BC based on molecular classification has been investigated and although appear promising, it is currently not mature enough for routine clinical application ( 13 ). There are three risk groups of BC, based on predicted recurrence and progression rate derived from the European organization for research and treatment of cancer (EORTC) ( 13 ). Low-risk (LR) BC include primary, solitary, pTaG1 (PUNLMP), <3cm, no Cis; high-risk (HG) include pT1, G3 (HG), Cis, multiple, recurrent and >3cm pTaG1-2/LG tumours; and intermediate-risk include tumours not defined in the low and high-risk groups. A subgroup of highest-risk tumours includes G3pT1/HG with Cis, multiple and/or large G3pT1/HG and/or recurrent G3pT1/HG, G3pT1/HG with prostatic urethra Cis and some forms of variant histology of urothelial carcinoma and LVI.

### EAU diagnostic guidelines prior to COVID-19

The European Association of Urology (EAU) recommend investigating BC with urinary cytology, CT urogram, flexible-cystoscopy and transurethral resection of the bladder tumour (TURBT), the latter can be both diagnostic and therapeutic for NMIBC ( 12 , 14 ). Urinary molecular markers such as UroVysion (FISH), Nuclear Matrix Protein 22 (NMP) and fibroblast growth factor receptor 3 (FGFR)/telomerase reverse transcriptase (TERT) have not been accepted for diagnosis or follow up in routine practice or clinical guidelines. Confirmed MIBC should be staged with CT thorax-abdomen-pelvis (TAP).

### EAU diagnostic recommendations during COVID-19

The EAU categorised diagnoses into four priority groups, defined as the following ( 15 ):

1. Low priority, clinical harm (progression/metastasis) very unlikely if service postponed by 6 months.
2. Intermediate, clinical harm possible if postponed 3-4 months, but unlikely.
3. High, clinical harm and cancer-related deaths very likely if postponed >6 weeks.
4. Emergency, life-threatening situation on opioid-dependent pain.

### NMIBC

LG NMIBC has a low cancer-specific mortality rate of around 1-2%, therefore, active surveillance is an appropriate management option ( 16 ). 1) low priority diagnostics can be deferred by 6 months; 2) intermediate priority, diagnosed before end of 3 months; 3) high priority, diagnosed within <6 weeks which include CT urogram and USS in patients with visible haematuria (VH) and cystoscopy in patients with VH without clots; 4) emergency, diagnosed within <24 hours which include TURBT in patients with VH and clot retention requiring bladder catheterization ( 15 ).

### MIBC

The diagnosis of low priority cases can be deferred by 6 months and intermediate priority cases before the end of 3 months. High priority cases should be diagnosed within <6 weeks and include MIBC staging with CT TAP.

### Alternatives

Although current diagnostic tools include urinary cytology, imaging, flexible-cystoscopy and TURBT, this may be the time to utilize molecular markers and next-generation sequencing to aid in the diagnosis and predicted outcome of NMIBC ( 17 , 18 ).

### EAU treatment guidelines prior to COVID-19

The management of BC is based on histology, grade and stage, patient's co-morbidities and performance status and patient's preference. NMIBC are given a single mitomycin instillation preferably in the first few hours following TURBT. Some histological confirmed tumours are subject to re-resection such as, HG, pT1, incomplete or no muscle obtained in the first resection ( 19 ). Following TURBT, low-risk NMIBC can be managed with cystoscopic surveillance at 3 and 12 months after diagnosis followed by annual cystoscopies for five years. High-risk NMIBC have the option of Bacillus Calmette-Guérin (BCG) intravesical instillations or radical cystectomy (RC) ( 20 ). MIBC are managed with cisplatin-based neoadjuvant chemotherapy (NAC) followed by RC and pelvic lymph node dissection (PLND) or bladder-sparing modalities including radiotherapy and chemo-therapy as part of a multimodal treatment ( 21 ). Metastatic BC are managed with cisplatin-based chemotherapy such as gemcitabine, cisplatin (GC), methotrexate, vinblastine, adriamycin, cisplatin (MVAC), paclitaxel, cisplatin, gemcitabine (PCG) and/or immunotherapy with checkpoint inhibitors (programmed death ligand 1).

### EAU treatment recommendations during COVID-19

### NMIBC

Low priority cases can be deferred by 6 months and include: 1) TURBT in patients with small papillary recurrence/s, <1cm and pTa/1 LG tumours, re-resection in patients with visibly complete initial TURBT of pT1 lesion with muscle in the specimen; 2) early post-operative chemotherapy instillation in presumably low/intermediate-risk tumours; 3) intravesical BCG or chemotherapy instillations in patients with intermediate-risk NMIBC ( 15 , 16 ).

Intermediate priority cases should be treated before the end of 3 months and include: 1) TURBT in patients with any primary tumour or recurrent tumour >1cm without VH or history of HR-NMIBC; 2) immediate RC in patients with highest-risk NMIBC; 3) early RC in patients with BCG unresponsiveness or failure ( 15 , 16 ).

High priority cases should be treated within <6 weeks and include: 1) TURBT in patients with bladder lesion and intermittent VH or a history of HR-NMIBC; 2) re-resection in patients with visibly residual tumour after initial TURBT and large or multiple HGpT1 at initial resection without muscle in the specimen; 3) induction intravesical BCG ± first maintenance therapy (6 + 3) in patients with HR-NMIBC. HR-NMIBC progress to muscle invasion or metastatic disease in 15-40% of patients and 10-20% may die from BC. Therefore, BCG is the preferred choice for most patients and maintenance therapy can be resumed when COVID-19 subsides ( 15 , 16 ). Emergency priority cases should be treated within <24 hours and include TURBT in patients with VH with clot retention requiring bladder catheterization ( 15 ).

### MIBC

Prolonged delays (>90 days) between TURBT and RC are associated with poor survival. Russell et al. found a significant risk of death for patients in which treatment was delayed (HR 1.34, 95%CI 1.18-1.53) ( 22 ). Lin-Brande et al. explored patients with variant histology undergoing RC and reported a significant increase in the risk of death in patients in whom surgery was delayed beyond 12 weeks (HR 3.45, 95% CI 1.51–7.86) ( 23 ). Kulkarni et al. reported a rise in the risk of death when there was a delay of >40 days between TUR and radical cystectomy ( 24 ). Although in patients who undergo NAC the delay between diagnosis and RC becomes less significant, the time between NAC and surgery has been explored as a risk factor for mortality. Boeri et al. reported a decreased survival for patients in whom this time frame was >10 weeks, with a 3-year survival of 64% vs 42% for patients operated, 10 weeks and >10 weeks respectively ( 25 ). Moreover, delay in surgery has been associated with an increased risk of upstaging ( 26 , 27 ). Thus, EAU guidelines recommend RC to be performed within 12 weeks. Therefore, during the pandemic, RC delays for MIBC of up to 12 weeks may be safe.

In low priority cases, consider omitting NAC (cisplatinum-eligible only) in T2-3 focal N0M0 patients. The proven benefit of NAC on T2 disease has to be weighed against the risks ( 15 ).

Intermediate priority cases should be treated before the end of 3 months and include: 1) offering RC in T2-4a, N0M0 tumours; 2) multi-modal bladder-sparing therapy can be considered for selected T2N0M0 patients; 3) chemoradiation should be offered to improve local control in cases of inoperable locally advanced tumours. In cT4 or N+, radical chemoradiation can be offered accepting that this may be palliative rather than curative in outcome ( 15 , 16 ).

High priority cases should be treated within <6 weeks and include: 1) TURBT for suspicious invasive tumour identified on imaging; 2) consider alternatives such as radiotherapy ± chemotherapy to palliative RC; 3) NAC for individualize risk in high burden T3-4 N0M0 patients while they are on the waiting list; 4) offer adjuvant cisplatin-based combination chemotherapy to patients with T3-4 and/or pN+ disease if no NAC was given ( 15 , 16 ).

Emergency cases include: 1) radiotherapy ± chemotherapy for intractable haematuria with anaemia; 2) nephrostomy for locally advanced BC with acute renal failure; 3) embolization or haemostatic radiotherapy for bleeding with haemodynamic repercussion ( 15 ).

Surgeons must consider that RC is a morbid surgery, with a risk of transfusions of 5-25% ( 28 ), as well as a non-negligible risk of Clavien IIIb complications ( 29 ) requiring further operating room occupation and eventual need for intensive care. Clearly, in times when intensive care units may be fully occupied during the COVID-19 pandemic, one must ask whether RC can be safely performed.

A thorough discussion with the patient should be carried out concerning the type of urinary diversion. Orthotopic neobladder reconstruction has been systematically associated to increased hospital stay and post-operative complications ( 30 ). Thus, although each patient does require specific decision making, a trend towards increased implementation of non-continent urinary diversion is probable. Furthermore, minimally invasive surgery, and in particular robotic-assisted radical cystectomy (RARC) is increasingly being implemented across urology departments in the effort to reduce the significant morbidity of radical cystectomy. Although results are contradictory ( 31 ), randomized controlled trial exploring RARC with extracorporeal urinary diversion did not find a significant reduction of post-operative complications ( 32 ). However, supporters of the robotic approach claim that an intracorporeal diversion ( 33 ) may indeed impact positively on the patients' recovery after surgery, hence one could speculate that RARC with intracorporeal urinary diversion could be an intriguing solution during the pandemic. However, the safety of the surgical team during laparoscopic surgery must be kept in mind, adopting the adequate protective equipment for surgeons involved in RARC, managing correctly the insufflation and exsufflation of pneumoperitoneum and limiting this surgery to expert centers.

Trimodal therapy (TMT), consisting of complete TURBT, chemotherapy and radiotherapy is an interesting alternative to surgery in selected patients ( 21 ). Studies have demonstrated its equivalence to RC in terms of oncologic outcomes ( 34 ). At a first glance, one could support the superiority of TMT over surgery during the outbreak, given its improved safety and reduced risk of complications, need for transfusion or occupation of intensive care units. However, one must consider that TMT requires a complete TURBT, 40-46 Gy radiation therapy, platinum-based chemotherapy and frequently, an additional TUR under general anaesthesia before receiving tumour boost radiation therapy of 20 Gy. This accounts for multiple accesses to a tertiary referral center, with a consequent increased risk of exposure to COVID-19, in patients potentially immunosuppressed due to chemotherapy. Therefore, although its undeniable benefits in terms of morbidity, TMT does indeed increase the number of accesses and transportations of patients to hospitals, potentially overcoming its benefits.

Adjuvant chemotherapy has an uncertain clinical benefit ( 35 ). On the other hand, it is associated with immunosuppression and increased risk of infective complications and as such, it should be avoided in times of COVID19.

### Metastatic BC

Intermediate priority cases require assessment of risk and benefit. Asymptomatic patients with low disease burden can postpone treatment (8-12 weeks) under clinical surveillance. Treatment include: 1) cisplatin-based combination chemotherapy; with GC, MVAC, preferably with G-CSF, high-dose MVAC with G-CSF or PCG; 2) offering checkpoint inhibitors depending on PDL1 status; 3) offering checkpoint inhibitor to patients progressing during or after platinum-based combination chemotherapy.

High priority cases should be treated within <6 week: 1) G-CSF should be considered for symptomatic patients; 2) cisplatin-containing combination chemotherapy with GC, MVAC, preferably with G-CSF, high-dose MVAC with G-CSF or PCG; 3) offer checkpoint inhibitors depending on PD-L1 status ( 15 ).

A regime comprising GC with G-CSF rather than MVAC may be preferred due to the higher likelihood of neutropenia in patients receiving MVAC ( 36 ).

### EAU follow up guidelines prior to COVID-19

### NMIBC

Patients with LR pTa tumours should undergo cystoscopy at 3 and 12 months following diagnosis and yearly for 5 years. Patients with HR NMIBC should undergo cystoscopy every 3 months for 2 years, every 6 months until 5 years and then yearly thereafter. Patients with intermediate risk tumours should have an in-between (individualized) follow-up scheme. Rigid cystoscopy and bladder biopsies should be performed when check flexible cystoscopy shows suspicious findings. Those who are on maintenance BCG undergo interval check cystoscopies and biopsies ( 14 ).

### MIBC

Patients who underwent RC should have a CT scan every 6 months until the third year, followed by annual imaging thereafter to monitor for local and upper tract recurrences. Those who received radiotherapy should undergo cystoscopic surveillance as per the HR-NMIBC protocol ( 12 ).

### EAU follow up recommendations during COVID-19

### NMIBC

Low priority cases are deferred by 6 months and follow up include: 1) cystoscopy in patients with a history of low/intermediate-risk NMIBC without haematuria; 2) upper tract imaging in patients with a history of HR-NMIBC ( 15 ).

Intermediate priority cases should be followed up before the end of 3 months cystoscopy in patients with a history of HR-NMIBC without haematuria.

High priority cases should be followed up within <6 weeks with cystoscopy in patients with NMIBC and intermittent haematuria.

Emergency cases should be followed up within <24 hours with cystoscopy or TURBT in patients with VH with clots.

### MIBC

Routine follow up periods after RC should be extended to 6 months ( 15 ).

## CONCLUSION

During the COVID-19 outbreak it is safe to postpone surveillance and TURBT for low and intermediate risk BC. Patients presenting de novo haematuria should undergo urinary cytology, USS kidney-ureter-bladder or clinical cystoscopy to assess their risk status. BCG induction and one course of maintenance should be offered as first line treatment in patients with HG-NMIBC. Re-resection should be limited to more aggressive cases or when the risk of residual tumour is present. Higher risk cases should undergo RC if hospital capacity and COVID-19 burden allows. RC can be delayed by up to 12 weeks without causing harm to the patient. NAC should be considered balancing benefits from the therapy and risks for immunosuppression. TMT may have a potential role according to the facility of the hospital.
